# Changes in the corpus callosum during the recovery of aphasia: A case report

**DOI:** 10.1097/MD.0000000000011155

**Published:** 2018-06-15

**Authors:** Qiwei Yu, Weixin Yang, Yi Liu, Hong Wang, Zhuoming Chen, Jiajian Yan

**Affiliations:** a Department of Rehabilitation Medicine, The Affiliated Suzhou Hospital of Nanjing Medical University, Suzhou; b Department of Rehabilitation Medicine, The First Affiliated Hospital of Jinan University, Guangzhou, China.

**Keywords:** corpus callosum, diffusion tensor imaging, diffusion tensor tractography, poststroke aphasia

## Abstract

**Rationale:**

The corpus callosum, which is the most important fiber pathway linking the bilateral hemispheres, plays a key role in information access, as well as the functional coordination and reorganization between the bilateral hemispheres. However, whether the corpus callosum will undergo structural changes during the recovery of aphasia is still unclear. In the current study, a Chinese aphasic patient with stroke was reported to develop changes in the corpus callosum after speech therapy.

**Patient concerns:**

A 33-year-old right-handed male patient had aphasia only without limb paralysis at 14 months after stroke.

**Diagnoses:**

Neuroimaging evaluation confirmed a diagnosis of cerebral infarction in the left frontal lobe, insula and basal ganglia.

**Interventions:**

He underwent 5-month speech therapy and received language function evaluation and DTI examination before and after speech therapy.

**Outcomes:**

The result ABC showed that the language functions in the patient, including spontaneous speech, auditory comprehension, repetition and naming, were improved after the speech therapy. In addition, results of follow-up DTT suggested that the fiber pathway between the splenium of corpus callosum and the left superior temporal gyrus (Wernicke's area) had been established. At the same time, fiber connections between the genu of corpus callosum and the right inferior frontal gyrus (the mirror region of Broca's area) were increased.

**Lessons:**

The fibrous structure between the corpus callosum and cortical language areas may be reconstructed during the recovery of aphasia. In addition, and the corpus callosum may play an important role in the occurrence and recovery of aphasia after stroke.

## 1 Introduction

Aphasia is a common consequence of stroke; however, the mechanism of recovery has not been elucidated yet. Several mechanisms have been proposed currently, including the contribution of either the perilesional region in the affected hemisphere or the homologous language regions in the unaffected hemisphere.^[1,2]^ Thus, it is obvious that the bilateral hemispheres of brain are critical for the recovery of aphasia. In addition, a growing number of literature has pointed out that the white matter tracts play a crucial role in understanding the neural mechanisms of language processing, and determining the nature of language deficits and recovery patterns in aphasia.^[3]^ Typically, the corpus callosum, which is the most important fiber pathway linking the bilateral hemispheres, plays a key role in information access, as well as the functional coordination and reorganization between the bilateral hemispheres. However, whether the corpus callosum will undergo structural changes during the recovery of aphasia is still unclear. In the present study, a Chinese aphasic patient with stroke was reported to develop changes in the corpus callosum after speech therapy.

## 2 Case description

**Figure 1:**
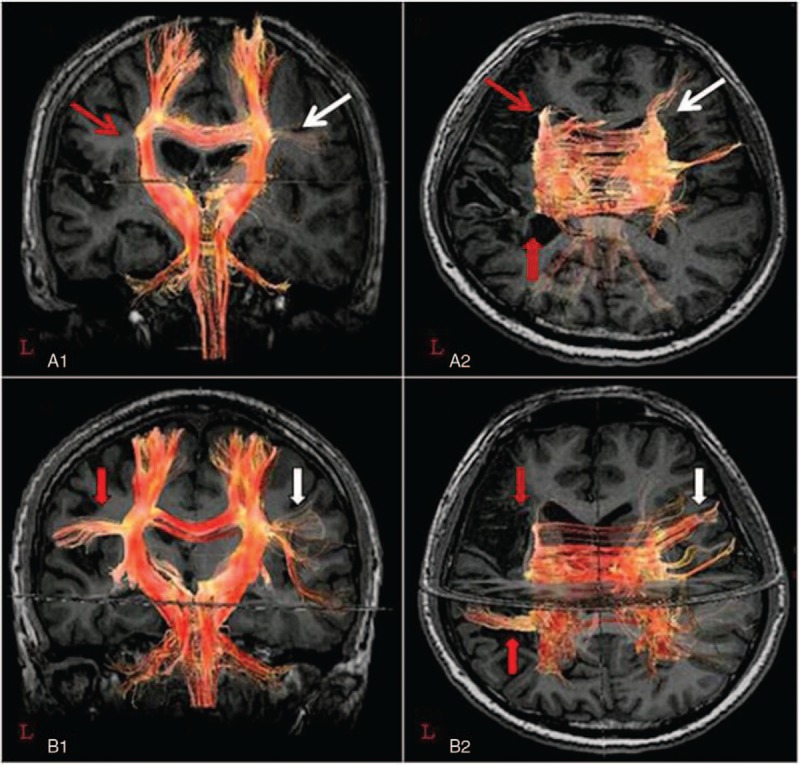
DTI fiber tracking of the corpus callosum before (A) and after (B) speech therapy.

The 33-year-old right-handed male patient, with no history of brain damage had suffered from a stroke. Brain magnetic resonance imaging (MRI) revealed an infarction lesion in the left frontal lobe, insula, and basal ganglia (Fig. 1A). 14 months after stroke, the patient had aphasia only without limb paralysis; thus, he received a 5-month speech therapy composed of spontaneous speech, auditory comprehension, repetition, naming, writing, reading training, and calculation. The speech therapy was carried out for 1 hour per session, for twice a day and 5 days a week. Moreover, the Aphasia Battery of Chinese (ABC) and Boston Diagnostic Aphasia Examination (BDAE) were used to evaluate the language function and the severity of aphasia before and after the speech therapy. Briefly, ABC is a modified Western Aphasia Battery (WAB) adapted to the Chinese culture, which is currently the most extensively used scale in China for aphasia assessment, with well-established reliability and validity.^[4]^ Informed consent was given and the experiment was approved by the Ethics Committee of Jinan University First Affiliated Hospital in Guangzhou, China.

The results suggested that the patient had attained certain improvements in his language function (spontaneous speech: 69.8 percentile, auditory comprehension: 66.4 percentile, repetition: 64.0 percentile, and naming: 84.8 percentile). Besides, the severity of aphasia was rated at level 2 before the speech therapy. After the speech therapy, further language functional improvements were achieved (spontaneous speech: 81.1 percentile, auditory comprehension: 69.4 percentile, repetition: 72.0 percentile, and naming: 84.8 percentile), and the severity of aphasia was rated at level 3.

### 2.1 Diffusion tensor imaging

Diffusion tensor imaging (DTI) was carried out before and after the speech therapy, respectively. In addition, an 8-channel head coil equipped on a 3.0 T American GE Discovery 750 MRI with single shot echo-planar imaging was used for the acquisition of DTI data. A total of 47 contiguous slices parallel to the anterior commissure–posterior commissure line was acquired for each of the 25 noncollinear diffusion sensitizing gradients. Specifically, the imaging parameters were as follows: TR/TE = 5000 ms/68.0 ms, acquisition matrix = 96 × 96, reconstructed to matrix = 128 × 128 matrix, field of view = 25.6 × 25.6 mm^2^, *b* = 1000 s / mm^2^, NEX = 1, slice thickness/slice spacing = 3 mm/0 mm. It took 135 seconds for DTI data acquisition. Besides, the corpus callosum was tracked using the self-prepared software package (Functool 9.4.05a). Fiber tracking was initiated at the center of a seed voxel with a fractional anisotropy (FA) of >0.18, apparent diffusion coefficient of >0.01, and the maximum steps of 160.

Results of the follow-up DTI fiber tracking demonstrated that the fiber pathway between the splenium of corpus callosum and the left superior temporal gyrus (Wernicke's area) had been established. In addition, the fiber connections between the genu of corpus callosum and the right inferior frontal gyrus (the mirror region of Broca's area) were increased (Fig. 1B).

## 3 Discussion

Anatomically, the anterior 1/3 of the corpus callosum is connected to the left motor language area, whereas the posterior 1/3 is connected to the left sensory language area and the right motor language mirror area. Functionally, the corpus callosum is closely related to the language function.^[5,6]^ Several studies^[7,8]^ have suggested that corpus callosum injury would lead to aphasia. However, the precise brain mechanism of aphasia has not been illustrated in these studies. Ishizaki et al^[9]^ had reported a patient with crossed aphasia resulted from infarction of the right corpus callosum, and suspected that transcallosal diaschisis was the possible mechanism of aphasia. Physiologically,^[10,11]^ the excitability between the bilateral cerebral cortices is in equilibrium, which can be attributed to the presence of transcallosal inhibition. However, the suppression on the right hemisphere has diminished or even disappeared when the left hemisphere language area is damaged, which is the result of the relieved “transcallosal inhibition.” This will thus result in the relatively increased excitability in the right hemisphere language mirror area. In other words, damage in the left hemisphere language area will give rise to diaschisis between the bilateral cerebral cortices. In this study, apart from the damage in the left hemisphere, the transcallosal diaschisis in the primary language areas may account for the possible mechanism leading to aphasia in the patient.

Recovery of aphasia is a process to re-establish the excitement balance between the bilateral hemispheres. Notably, the right-language mirror area, as well as the remaining left-language area and neighboring area, can influence each other, jointly promote the reorganization of language networks, and compensate for the impaired language functions.^[12]^ Therefore, the corpus callosum is suggested in some studies^[13–15]^ to play a critical role in the recovery of aphasia. The corpus callosum may undergo structural and functional changes during the recovery of aphasia, which can thereby promote the structural and functional reorganization of the language network. Our findings indicate that, during the recovery of aphasia, the corpus callosum has undergone a structural change, which may lead to the re-establishment of the excitement balance between the bilateral brain hemispheres as well as the effective reorganization of the impaired language network. This may account for one of the important mechanisms for improving the spontaneous speech and repetition ability of patients. Moreover, the discontinuation of fiber connections between the left Broca and Wernicke areas may be one of the causes of the slightly improved auditory comprehension and naming ability in the patient.

In conclusion, the recovery mechanism of the speech function in our patient remains unclear. Nevertheless, our results have preliminarily suggested that, during the recovery of aphasia, the fibrous structure between the corpus callosum and cortical language areas may be reconstructed. In addition, the corpus callosum may play an important role in the occurrence and recovery of aphasia after stroke. Such findings are of great significance, which can provide scientific support for the necessity of clinical language rehabilitation therapy. Consequently, in the case of expanding sample size, further complementary studies employing functional MRI and DTI are required in the future, so as to examine the functions of the cerebral cortex and the structure state of the white matter fiber tracts.

## Author contributions

**Conceptualization:** Zhuoming Chen, Jiajian Yan.

**Funding acquisition:** Hong Wang.

**Investigation:** Qiwei Yu.

**Methodology:** Qiwei Yu.

**Project administration:** Hong Wang.

**Resources:** Hong Wang.

**Software:** Qiwei Yu.

**Validation:** Weixin Yang, Yi Liu.

**Writing – original draft:** Qiwei Yu.

**Writing – review and editing:** Hong Wang.
